# Open Air Treatment of Influenza

**Published:** 1919

**Authors:** 


**The Open Air Treatment of Influenza.** By William A. BROOKS. *The American Journal of Public Health,* October 1918.

When this influenza broke out, the autopsies at the Naval Hospital showed that the men died not from heart failure, but from abscesses in the lungs. This explained why heart stimulants had had practically no effect on the cyanosed patients, and that they died not from heart failure but from lack of air.

Thereafter, on pleasant days, every patient was taken out of the tents at the Tent Hospital which had been rapidly set up. From the first day the results were startling.

At the end of 3 weeks the epidemic on the ships was practically under control, and in a few days over four weeks the Tent Hospital was closed.

The following facts were deduced from the experiences of the first tent hospital :

The period of incubation apparently varies, averaging about two days. Fatigue seemed to play an important part.

The infection itself was a mixed infection, and where Type III of the pneumococcus predominated many of the cases were fatal.

Very little medicine was given after the value of plenty of air and sunshine had been demonstrated. Practically only three drugs were used : Dover's powder, some form of aspirin, and iodide of lime in one-third-grain doses. The patients were fed every 2 or 3 hours, and given a variety of food with plenty of fruit. They were also made to drink plenty of water.

Improvised wire masks were made out of ordinary gravy strainers. The strainer was made to fit the nose, and 5 layers of gauze were simply basted on to the wire frame. The gauze on the masks was changed every 2 hours.

Every nurse and attendant was cautioned that, after working over patients, the hands should be washed thoroughly either in 1/1000 corrosive sublimate solution, or in a solution of triple lysol.

The efficacy of open-air treatment has been absolutely proven, and one has only to try it to discover its value.

